# Individual and Synergistic Effects of Hybrid PVA–Steel Fiber on Mechanical Properties of Nano-SiO_2_ Modified Epoxy Resin Gel Mortar

**DOI:** 10.3390/gels12050424

**Published:** 2026-05-12

**Authors:** Peng Zhang, Xiao Zhang, Xiaobing Dai, Shiyao Wei

**Affiliations:** School of Water Conservancy and Transportation, Zhengzhou University, Zhengzhou 450001, China; zhangpeng@zzu.edu.cn (P.Z.); 15690862775@163.com (X.Z.); wsy1124jkl@163.com (S.W.)

**Keywords:** hybrid fiber, mechanical properties, toughness, bonding properties, SEM analysis

## Abstract

Nano-SiO_2_-reinforced epoxy resin gel mortar (NERM) serves as an essential material for repairing and strengthening defective structures in civil engineering. This study developed a hybrid fiber-reinforced NERM (HF-NERM) by incorporating PVA–steel fiber, aiming to achieve superior mechanical properties, toughness, and bonding performance. This study systematically investigates the workability, mechanical properties, toughness, and bonding characteristics of HF-NERM, as well as their enhancement mechanisms characterized using scanning electron microscopy (SEM). Experimental results indicate that the slump of HF-NERM decreased significantly with increasing hybrid fiber content, and the regression coefficient of PVA fiber on slump was −86.7, while that of steel fiber was −4.5. The addition of hybrid fibers generally enhanced the mechanical properties. The optimal combination was 0.9% PVA fiber and 1.2% steel fiber, at which the flexural strength reached 11.56 MPa with an increase of 32.57%, splitting tensile strength reached 4.42 MPa with an increase of 20.1%, and interfacial bonding strength was improved by 9.8%. With the exception of splitting tensile strength, most mechanical properties initially increased and then decreased with increasing hybrid fiber content, indicating an optimal dosage. The hybrid fibers also enhanced the flexural toughness of HF-NERM; the toughness indices I_5_, I_10_ and I_20_ were increased by 20.99%, 24.12% and 65.83%, respectively, and the residual strength factors R_5,10_ and R_10,20_ were increased by 26.8% and 160.8%. The hybrid fibers also enhanced the flexural toughness of HF-NERM. Mechanistically, PVA fibers primarily contributed to preventing the development of micro-cracks, while steel fibers were the main contributors to resisting macro-cracks. SEM observations demonstrated that the failure modes of PVA fibers involved synergistic mechanisms, while those of steel fibers were relatively singular. Related enhancement mechanisms were discussed based on the experimental results. Finally, the results demonstrate that NERM could be effectively strengthened by adding an appropriate content of hybrid fibers. This study’s novelty lies in quantifying the individual and synergistic effects of PVA–steel fibers in the NERM system, establishing optimal dosage parameters, and revealing matrix–fiber interaction mechanisms specific to epoxy-based composites. The findings provide a reliable material design basis for high-performance repair mortars and offer practical guidance for extending the service life of aging civil engineering structures.

## 1. Introduction

Cementitious composites are widely employed in construction due to their remarkable mechanical properties and durability. However, with increasing service life, many concrete structures are aging, necessitating maintenance and reinforcement for a significant number of them. Nano-SiO_2_-reinforced epoxy resin gel mortar (NERM), offering high mechanical properties and excellent workability, has been extensively applied in repairing existing structures [1,2,3]. Nevertheless, similar to conventional cementitious composites, NERM exhibits low flexural strength and poor crack resistance. Therefore, it is essential to find effective methods to enhance the mechanical performance of NERM.

Fiber reinforcement is a well-established technique to address such limitations in cementitious materials. Fiber-reinforced cementitious composites (FRCC) are typically composed of cementitious matrices reinforced with discontinuous short or continuous long fibers [4]. Extensive research over the past decades has demonstrated that incorporating fibers enhances crack resistance, flexural toughness, strength, and durability [5,6,7,8,9]. However, current investigations and applications of FRCC primarily focus on ordinary Portland cement (OPC) systems. Comparatively, fewer studies have been conducted on fiber-reinforced NERM. For instance, Ahirwar found that incorporating recycled glass fibers and bamboo fibers significantly improved mechanical properties [10]. And Luo et al. reported that polyvinyl alcohol (PVA) fiber exhibited excellent bonding properties with the epoxy-resin-modified cementitious matrix [11]. Importantly, FRCC is a typical multiphase and multi-scale composite material, resulting in a multi-stage failure process [12,13]. The properties achievable with a single type of fiber are often limited and may not meet the comprehensive requirements for reinforcing cementitious composites. Consequently, the use of hybrid fibers has been extensively employed to improve composite performance [14,15]. Hybrid fibers can be classified into high and low elastic modulus types based on their mechanical properties. High elastic modulus fibers primarily enhance the strength of the matrix, while low elastic modulus fibers increase deformation capacity. In cementitious composites, steel fiber and PVA fiber are characteristic representatives of high and low elastic modulus fibers, respectively [13,16]. Despite these valuable findings, the reinforcement mechanisms and optimal parameters derived from cementitious systems cannot be directly transposed to NERM. This is because NERM features a non-hydration curing process, a dense organic–inorganic composite microstructure, and distinct interfacial adhesion mechanisms between fibers and the epoxy–silica matrix, leading to unique fiber–matrix interaction behaviors.

Steel fiber is a kind of fiber with extremely excellent performance, featuring high strength and elastic modulus [17]. When steel fiber is incorporated into cementitious composites, it can prevent the development and expansion of macro-cracks of the materials, thereby significantly enhancing the mechanical properties of FRCC [18,19]. It has been demonstrated that the incorporation of steel fiber improves the mechanical properties of FRCC [20]. For example, the compressive properties of ultra-high-performance FRCC at 7 days was all elevated (18.56–42.31%) under each dosage of steel fiber [20]. The results of Mohammadi et al. illustrate that the length-to-diameter ratio of steel fiber exhibited a significant impact on the workability of the mixture [21]. It was noted by Akcay and Tasdemir that the workability of fresh concrete was only slightly adversely affected by a high dosage of steel fiber [22]. PVA fiber is a synthetic fiber processed from polyvinyl alcohol, which exhibits high strength, excellent affinity and adhesion with cement, good dispersibility and alkali resistance and, furthermore, it is harmless to the environment [11,23,24]. Therefore, PVA fiber was extensively employed in FRCC for decades. Specifically, the incorporation of PVA fiber significantly improved the compressive strength, flexural strength and toughness of FRCC [25,26,27,28].

Available studies provide valuable achievements that illustrate a deep understanding of the impact of fiber in FRCC. However, most previous studies mainly concentrated on conventional cement-based matrices, and the mechanical performance evolution, synergistic mechanism and interfacial microstructure characteristics of HF-NERM have not been systematically explored. The lack of quantitative design parameters and internal toughening mechanism analysis seriously restricts the engineering application of HF-NERM in structural repair and reinforcement. Accordingly, this work aims to explore the individual and synergistic influence of PVA–steel fiber on the mechanical properties and toughness of HF-NERM. Scanning electron microscope (SEM) was used to understand the failure form of fibers within the matrix. By comparing with findings from traditional cement-based hybrid fiber composites, this study not only verifies the adaptability of PVA–steel hybrid reinforcement to the epoxy-based matrix but also reveals unique enhancement mechanisms specific to NERM. Meanwhile, related mechanisms were discussed based on the experimental results. The results obtained from this study will provide a promising design solution for HF-NERM which can be used for existing concrete structure repair, and also supplement the basic experimental data and theoretical reference for the application of hybrid fibers in epoxy resin gel mortar composite systems, distinguishing this work from routine incremental research on traditional cementitious materials.

## 2. Results and Discussion

### 2.1. Fluidity Properties

**Figure 1:**
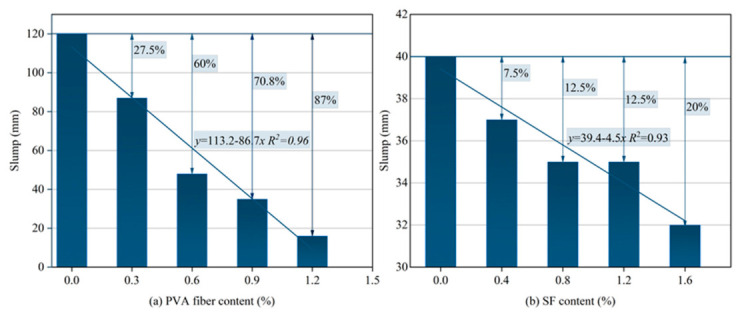
Effect of (**a**) PVA fiber and (**b**) steel fiber dosage on slump of HF-NERM.

The slump test results of HF-NERM influenced by PVA fiber and steel fiber are illustrated in Figure 1. It is obvious that with the incorporation of PVA and steel fiber, the slump of HF-NERM declined, while the reduction rate impacted by PVA fiber was bigger than steel fiber. The relationship between the slump of HF-NERM and PVA fiber was linearly fitted. The results illustrate that the regression coefficient of PVA fiber on slump was −86.7, while that of steel fiber on slump was −4.5. The reason for this phenomenon was mainly attributed to the surface discrepancy between PVA fiber and steel fiber. The surface of PVA fiber had a hydroxy group which was hydrophilic while the surface of steel fiber was coated with copper which was inert. Secondly, compared to steel fiber, the PVA fiber was soft and had a bigger length–diameter ratio which made the PVA fiber easily form the net structure during the mixing process.

### 2.2. Compressive Properties

**Figure 2:**
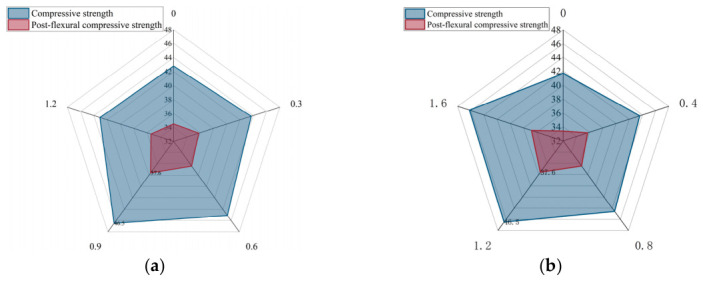
Impact of (**a**) PVA fiber and (**b**) steel fiber content on compressive properties of HF-NERM.

Figure 2 presents the compressive properties of HF-NERM influenced by PVA fiber and steel fiber. Statistical analysis was performed to evaluate the significance of differences between groups. The results indicate that the compressive strength at the optimal dosage was significantly higher than that of the control group and other fiber-content groups. Experimental results illustrate that the optimal dosage of PVA fiber is 0.9%, and for steel fiber it is 1.2%. In addition, based on Equation (1), when the incorporations of PVA fiber and steel fiber were 0.9% and 1.2%, the hybrid effect index was 0.0985. Compared to the other hybrid fiber researched by other scholars, it exhibited a better hybrid effect [29]. This indicates that steel fiber and PVA fiber have an excellent mixing effect. For this phenomenon, there were several reasons. First, PVA fibers can prevent cracks in specimens caused by lateral deformation and reduce the degree of stress concentration at the tip of the crack [30]. For steel fibers, the increase in the content of steel fibers lead to the formation of an overlap between the steel fibers and the matrix. The joint structure was stable and exerted a great binding force on the matrix, thereby enhancing the compressive strength. However, an extensive dosage of steel fiber and PVA fibers would decrease the compressive performance of HF-NERM. When the fiber content was too high, the steel fibers would tangle with the PVA fibers, causing agglomeration and resulting in compressive strength reduction.

### 2.3. Flexural Properties

#### 2.3.1. Flexural Strength

**Figure 3:**
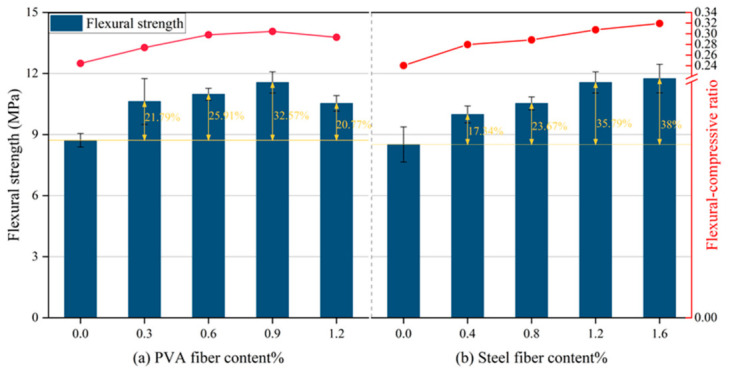
Impact of fiber on flexural strength and flexural–compressive ratio of HF-NERM.

As shown in Figure 3, the flexural strength of HF-NERM initially increased and then decreased with the addition of PVA fibers. Statistical analysis confirmed that the peak value at 0.9% PVA fiber was significantly higher than that of the control group (*p* < 0.05). When the PVA fiber content reached 0.9%, the flexural strength of HF-NERM peaked at 11.56 MPa, representing a 32.57% increase compared to the group without PVA fibers. This improvement could be attributed to hydroxyl groups on the PVA fiber surface, which formed hydrogen bonds with the cement matrix. Additionally, the irregular cross-sectional shape of PVA fibers enhanced the bonding area between fibers and the cement matrix, creating robust hydrogen-bonded bridges that effectively impeded and delayed crack propagation [31,32]. However, when the PVA fiber content increased to 1.2%, the flexural strength of HF-NERM declined. This reduction is likely due to excessive fiber agglomeration within the matrix, which introduced additional internal defects [24].

As illustrated in Figure 3, with the incorporation of steel fibers, the flexural strength of HF-NERM exhibited a tendency of continuously increasing. When the steel fiber content was 1.6%, the flexural strength of HF-NERM reached the highest value which was 11.75 MPa. Compared to the group without steel fibers, the increasing rate was 38%. It demonstrates that the incorporation of steel fibers had an excellent effect on the flexural strength of HF-NERM. It was mainly attributed to the bridging effect of steel fibers, which could effectively prevent the development of cracks [33]. Based on Equations (1) and (2), when the incorporation of PVA fibers and steel fibers was 0.9% and 1.2%, the αsp was 0.37. It demonstrates that the PVA fiber and steel fiber had an excellent hybrid effect. Figure 3 shows the flexural–compressive ratio of HF-NERM impacted by PVA fiber and steel fiber. The change trend of the flexural–compressive ratio illustrates that the addition of PVA fiber and steel fiber has played a good role in improving the toughness and crack resistance of HF-NERM.

#### 2.3.2. Flexural Toughness

**Figure 4:**
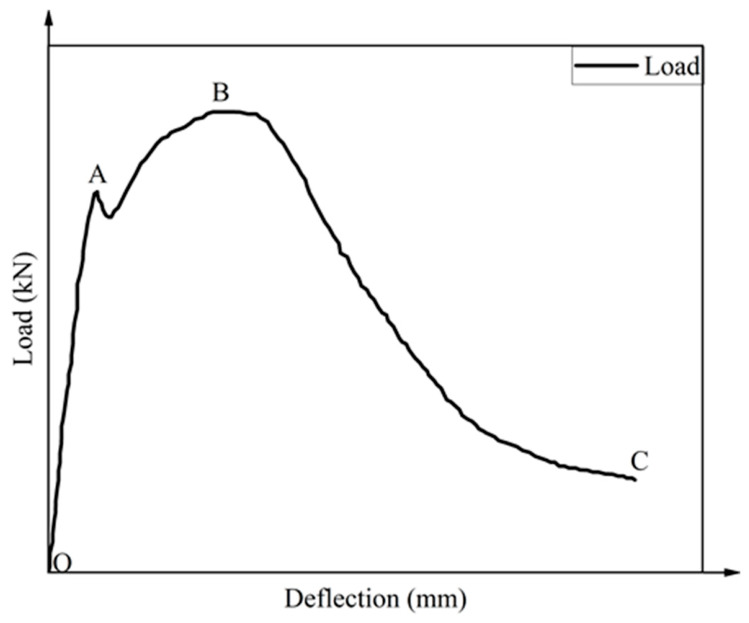
Typical load–deflection curve of HF-NERM.

Based on a four-point flexural test, a typical load–deflection curve was drawn, as shown in Figure 4. Generally, a typical load–deflection curve is divided into three stages:

(1) Section OA: At this stage, the curve follows Hooke’s Law, and the load increased linearly with the increase in mid-span deflection. At this stage, the hybrid fiber and the matrix bare the external force as a whole. At the end of the stage, micro-cracks appeared at the bottom of specimens and developed slowly.

(2) Section AB: At this stage, the curve changed to a nonlinear curve. The load first decreased rapidly. When it dropped to a certain value, the bridging effect between PVA fibers and steel fiber effectively prevented the load from further decreasing. The load gradually recovers and increases with the growth of deflection. However, the growth rate of deflection was faster than the load. At this stage, the micro-cracks inside the specimen steadily expanded. The matrix transported the stress to the fibers through interfacial bonding. Then the stress was transported to the uncracked matrix on both sides of the crack through bridging until the ultimate bearing capacity of the matrix was reached.

(3) Section BC: Following the peak load, the specimens entered a failure stage characterized by a progressive loss of bearing capacity (caused by fiber pull-out and fracture) and a concurrent acceleration in deflection growth, culminating in total failure.

**Figure 5:**
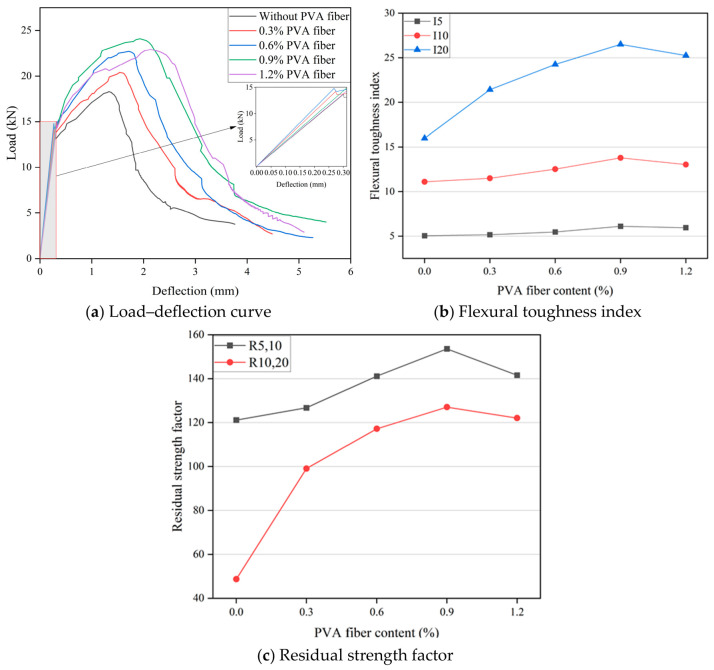
Effect of PVA fiber content on flexural toughness of HF-NERM.

**Figure 6:**
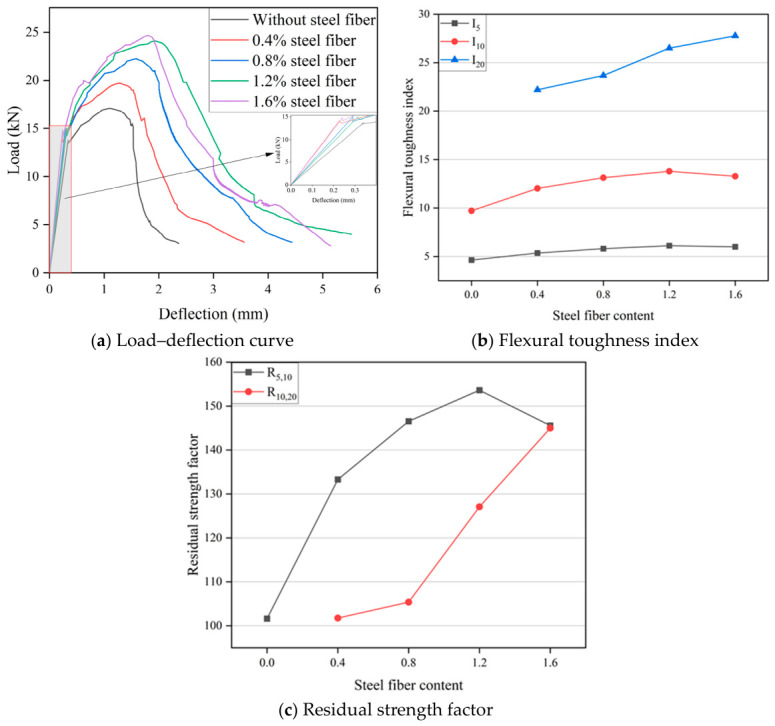
Effect of steel fiber content on flexural toughness of HF-NERM.

Flexural toughness of HF-NERM influenced by PVA fiber content and steel fiber content is illustrated in Figure 5 and Figure 6. It can be seen in Figure 5a that the load–deflection curve shows obvious strain-softening characteristics in the third stage of specimen failure when only the steel fiber is incorporated. With the addition of PVA fibers, the curve becomes fuller and the number of “plateau sections” increases. Meanwhile, the curve after the peak becomes flatter, compared to when only the steel fiber is incorporated. With the increase in deflection, the load shows an inconspicuous reduction. It can be concluded that the strain-hardening characteristics of the specimens become obvious. In contrast to the trend effect of the PVA fiber content, with the incorporation of steel fiber, the load–deflection curve becomes thinner and thinner. In addition, some individual specimens exhibit a sharking phenomenon on the load–deflection curve. It is mainly attributed to the damage and peeling of the matrix on both sides of cracks caused by the pull-out process of steel fiber.

Based on Equations (3)–(5), the flexural toughness index of HF-NERM influenced by hybrid fiber was calculated, as presented in Figure 5b and Figure 6b. When the specimen was added with 1.2% steel fiber, with the incorporating of PVA fiber content, the flexural toughness I5, I10 and I20 gradually increased. When the PVA fiber content was 0.9%, the toughness index I5 reached the maximum value. Compared with the only-steel-fiber-incorporated specimen, the increases were 20.99%, 24.12% and 65.83% respectively. With the continuous increase in the content of PVA fibers, the toughness indices all decreased. When the specimens were doped with 0.9% PVA fibers, with the increase in the steel fiber content, the toughness index generally showed a trend of rising first and then falling. I5 and I10 reached their maximum value at 1.2% steel fiber, and compared with when only PVA fibers were added, the increase rates were 31.97% and 42.02% respectively. While I20 reached its peak at 1.6% steel fiber, when compared with 0.3% steel fiber, the increase rate reached 25.13%. From the results of the experiment, the improvement of PVA fiber’s flexural toughness in the late stage of HF-NERM was significantly better than with steel fiber. In addition, the residual strength factor of HF-NERM influenced by PVA fiber and steel fiber is illustrated in Figure 5c and Figure 6c. R5,10 indicates that HF-NERM retained some of its interfacial bonding strength between hybrid fibers and the matrix, while R10,20 indicates that HF-NERM still retained the capacity to absorb flexural energy after instability [29]. When the PVA fiber content was 0.9%, the R_5,10_ and R_10,20_ of HF-NERM reached their maximum values, which were 26.8% and 160.8% higher than that of PVA fiber, which was 0%. This demonstrates that the incorporation of PVA fiber has a great capacity to absorb flexural energy after instability. Similar to the influence of PVA fiber, when the addition of steel fiber was 1.6%, the R10,20 of HF-NERM reached its maximum value, which was 42.8% higher than without steel fiber addition.

Four-point bending tests quantified mechanical parameters including initial cracking load, peak load, and ultimate deflection, as summarized in Table 1. For the initial cracking load of the HF-NERM, fixing steel fiber content at 1.2% yielded only a marginal 5.63% increase with PVA fiber content rising from 0% to 1.2%; similarly, fixing PVA fiber content at 0.9% resulted in merely an 8.45% increase as steel fiber content rose from 0% to 1.6%, indicating minimal influence of hybrid fiber content on initial cracking. Regarding peak load for HF-NERM, with steel fiber fixed at 1.2%, peak load initially increased before declining with rising PVA fiber content, peaking at 0.9% PVA fiber with a 34.41% increase relative to steel fiber alone; conversely, with PVA fiber fixed at 0.9%, peak load progressively increased with steel fiber content, maximizing at 1.6% steel fiber with a 50.61% increase over PVA fiber alone, demonstrating that hybrid fibers effectively enhance ultimate flexural capacity, where steel fibers were significantly more effective than PVA fibers. For ultimate deflection of HF-NERM, with steel fiber fixed at 1.2%, ultimate deflection increased progressively with PVA fiber content, peaking at 1.2% PVA fiber with a 76.81% increase over steel fiber alone; with PVA fiber fixed at 0.9%, ultimate deflection initially rose before decreasing with increasing steel fiber content, maximizing at 1.2% steel fiber with a 58.91% increase over PVA fiber alone, indicating that hybrid fibers significantly enhanced crack resistance and deformation capacity, with PVA fibers providing superior ductility improvement compared to steel fibers.

In addition to the calculated toughness indices and residual strength factors, the total energy absorption capacity of HF-NERM was further quantified using the area under the load–deflection curve up to a deflection of 10.5δ. For the PVA fiber series (with steel fiber fixed at 1.2%), the total energy absorption increased from 33.56 to 61.64 as the PVA fiber content increased from 0% to 0.9%, representing an increase of 83.7%. For the steel fiber series (with PVA fiber fixed at 0.9%), the total energy absorption increased from—(no data, due to brittle failure) to 60.79 at 1.2% steel fiber, then slightly decreased to 50.00 at 1.6% steel fiber. These results confirm that appropriate hybrid fiber incorporation significantly improves the total energy dissipation capacity of HF-NERM, while excessive fiber content leads to a reduction in energy absorption due to fiber agglomeration.

### 2.4. Splitting Tensile Strength

**Figure 7:**
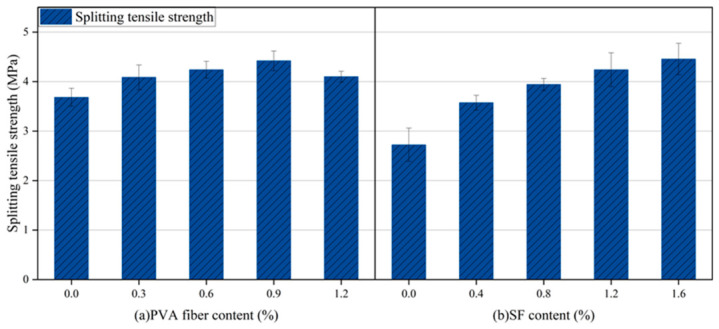
Splitting tensile strength of HF-NERM influenced by fibers.

Figure 7 illustrates how the content of PVA and steel fibers influences the splitting tensile strength of HF-NERM. Statistical analysis was carried out based on three parallel specimens for each group, with the error bars representing the standard deviation to ensure data reliability and repeatability. With the increase in PVA fiber content, the splitting tensile strength of PVA fiber exhibited a trend of first increasing and then decreasing. When the PVA content was 0.9%, the splitting tensile strength reached peak value at 4.42 MPa, which was 20.1% higher than when only steel fiber was incorporated. For the impact of steel fiber on splitting tensile strength of HF-NERM, the trend kept an increasing growth. The maximum splitting tensile strength of HF-NERM, representing a 54.86% increase over the none-steel-fiber, was achieved at a steel fiber content of 1.6%. The small standard deviations of all test results indicate good data reproducibility and statistically valid experimental outcomes. The above experimental results demonstrate that the addition of PVA fiber and steel fiber exhibits a significant improvement on splitting tensile strength of HF-NERM.

### 2.5. Interfacial Bonding Strength

**Figure 8:**
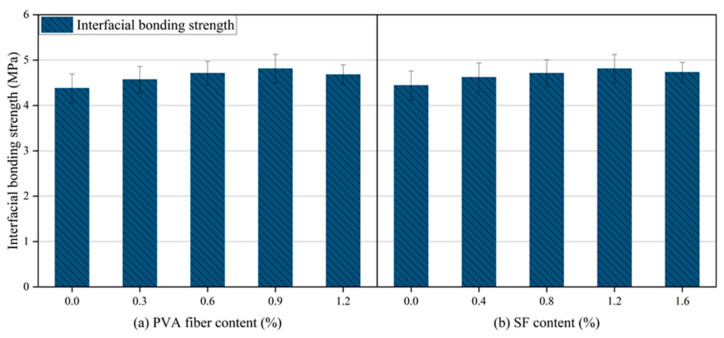
Interfacial bonding strength of HF-NERM impacted by fibers.

Figure 8 presents the bonding properties of HF-NERM influenced by hybrid fiber content. The interfacial bonding strength reached an optimum value of 4.81 MPa at a PVA fiber content of 0.9% (with steel fibers constant at 1.2%), representing a 9.8% improvement over the mix without PVA fibers. This value corresponds to the peak of an initial increase followed by a decrease in strength with increasing PVA content. For the incorporation of steel fiber, the interfacial bonding strength showed a similar trend as PVA fiber. When the steel fiber content was 1.2%, the interfacial bonding strength was max, which was 8.3% higher than without steel fiber content. Even though the percentage enhancement is modest, such an improvement in interfacial bonding is critical for repair mortar systems, as it directly determines the bonding reliability between the repair material and the existing concrete substrate. Sufficient interfacial bonding ensures effective stress transfer, prevents interfacial debonding or peeling failure, and thus enhances the long-term durability and service safety of repaired structures. In addition, the combined effect of PVA and steel fibers further stabilizes the interface transition zone, reduces internal defects, and improves the integrity between the fibers and the matrix. The experimental results of interfacial bonding strength demonstrate that the incorporation of PVA fiber and steel fiber had a significant hybrid effect within the matrix of NERM and exhibited remarkable improvement of bonding properties.

### 2.6. Related Mechanism

**Figure 9:**
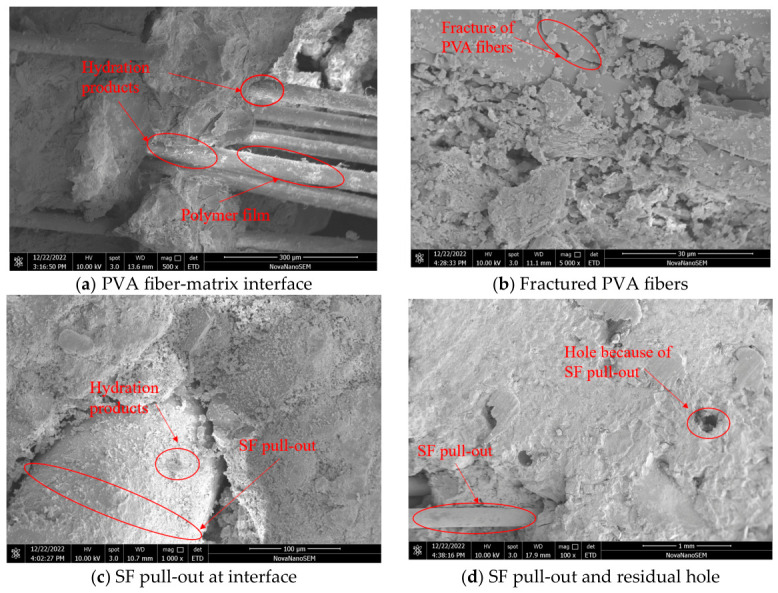
SEM observation of HF-NERM impacted by PVA fiber and steel fiber.

The mix theory of microfiber and macro-fiber can be employed to elucidate the above experimental results [34]. As a kind of microfiber, PVA fiber exhibits the ability to prevent the development of micro-cracks because of transverse deformation when specimens are subjected to external loads. Moreover, PVA can also reduce the degree of stress concentration at the crack tip [35]. The ability of PVA fiber to prevent the development of cracks is mainly attributed to the hydroxyl group on the surface of PVA fiber, which can react with the cement matrix and manufacture hydrogen groups [31]. Moreover, the irregular cross-section of PVA fibers expands the bonding surface between PVA fibers and the matrix, forming a firm hydrogen bridge between them and preventing and delaying the expansion of cracks [36]. In the matrix of HF-NERM, as shown in Figure 9a, the surface of PVA fibers is adhered with hydration products and polymer films, which is beneficial for the combination between PVA fibers and the matrix [12]. Figure 9b illustrates that the destructional form of PVA fibers within the matrix is not only pull-out but also fracture. This hybrid destruction form has been demonstrated to be efficient in improving the toughness and ductility of cementitious composites [37]. Consequently, the incorporation of PVA fiber has the ability to enhance the crack resistance, and increase the toughness and ductility of HF-NERM.

Steel fiber has been demonstrated to exhibit excellent improvement on bridging macro-cracks and enhance the toughness of cementitious composites [38]. On one hand, the bonding strength between steel fiber and the matrix effectively improves the expansion on cracks [39]. On the other hand, steel fiber has the function of bridging cracks and can effectively inhibit the development of cracks. As shown in Figure 9a,b, the destructional form of steel fiber is pull-out, and during the process of being pulled out, steel fiber can absorb a large amount of energy, thereby enhancing the mechanical properties of the matrix [8].

**Figure 10:**
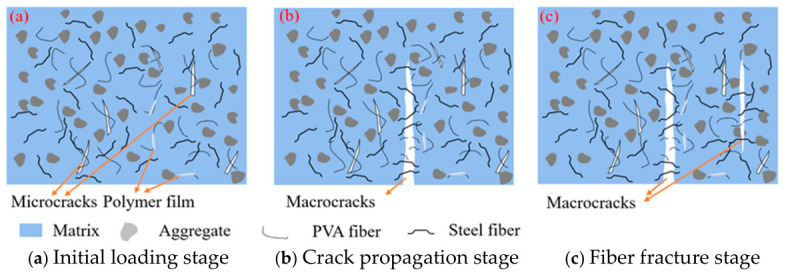
Enhance mechanism of hybrid fiber with the matrix of HF-NERM.

The mechanical properties of HF-NERM are significantly improved by adding PVA fiber and steel fiber. In the initial stage of increasing load, as shown in Figure 10a, PVA fibers bridge the microcracks, and prevent the development of microcracks to macrocracks, which further enhances the dissipation of energy [40]. PVA fibers play a leading role in inhibiting the propagation of microcracks when the microcracks develop to macrocracks. When the load increases to the peak value, the first macrocracks begin to appear, as shown in Figure 10b. Larger-sized steel fiber is difficult to break in HF-NERM and exhibits a pull-out effect, as shown in Figure 9c,d, which effectively suppresses macroscopic cracks and thereby enhances the toughness of HF-NERM [21]. As depicted in Figure 10c, when the steel fiber is going to be pulled-out, the interface between steel fiber and the matrix becomes loose and even damaged. However, the entanglement between PVA fiber and steel fiber inhibits the loosening and destruction of the interface between steel fiber and the matrix [41]. In HF-NERM, during the expansion stage of the macroscopic crack propagation process, steel fiber is the main factor hindering macroscopic crack propagation [42]. Therefore, the constraints of macroscopic and microscopic cracks lead to a significant increase in the strength and toughness of HF-NERM. The mixture of steel and PVA fibers achieves a synergistic reinforcing effect. It is worth noting that the improvement of ductility mainly relies on the synergistic mechanism of steel fiber. The deterioration in mechanical properties at high fiber contents is not only a macroscopic trend but also supported by microstructural evidence. Fiber agglomeration increases internal defects and reduces interfacial bonding strength, which directly explains the decrease in mechanical performance. This confirms that the optimal fiber dosage is determined by the balance between effective reinforcement and structural compactness.

## 3. Conclusions

The single and synergistic effects of PVA fiber and steel fiber on the mechanical properties of HF-NERM were investigated. The conclusions obtained from the experimental results are as follows:

(1) The slump of fresh HF-NERM decreases with increasing PVA fiber and steel fiber content, with PVA fiber exerting a far stronger negative effect on workability. Linear fitting yields a regression coefficient of −86.7 for PVA fiber (R^2^ = 0.96) and −4.5 for steel fiber (R^2^ = 0.93), confirming that PVA fiber significantly impairs fluidity more than steel fiber.

(2) An initial increase and then decrease trend in mechanical and bonding properties of HF-NERM was observed with the addition of PVA fiber and steel fiber. When the incorporation of PVA fibers and steel fiber was 0.9% and 1.2%, the hybrid effect index of compressive strength, flexural strength, splitting tensile strength and interfacial flexural strength was 0.0985, 0.372, 0.385 and 0.0917, respectively. The hybrid index indicates that the incorporation of hybrid fiber had an excellent effect on the mechanical properties of HF-NERM.

(3) Based on the load–deflection curve, the incorporation of hybrid fiber significantly improved the flexural toughness of HF-NERM. The addition of PVA fiber obviously effected the deformation capacity and steel fiber effectively enhanced the ultimate flexural bearing capacity. PVA–steel hybrid fibers could effectively improve the flexural toughness of HF-NERM. The optimal combination was when the specimens were doped with 0.9% PVA fiber and 1.2% steel fiber, and the flexural toughness indices were respectively greater than 5, 10 and 20 corresponding to their ideal elastoplastic materials.

(4) According to the SEM observation, the enhancing mechanism of PVA fiber and steel fiber on mechanical properties was established in this study. SEM experimental results demonstrate that the destructional forms of PVA fibers are pull-out and broken and for steel fiber it is pull-out. In the initial stage of load increase, the generation and development of microcracks were prevented by PVA fibers, and with the increasing of load, the presence of steel fibers inhibits the development of macroscopic cracks.

(5) This study supplements the experimental understanding of hybrid fiber modification for nano-SiO_2_-reinforced epoxy resin repair mortar. The developed HF-NERM exhibits a satisfactory strength level, favorable flexural toughness and stable interfacial bonding performance, showing good application potential for concrete structural repair scenarios with high requirements on crack resistance and durability. The present results provide experimental reference and basic design support for the optimization and engineering application of high-performance epoxy-based repair mortar reinforced with hybrid fibers.

## 4. Materials and Methods

### 4.1. Materials

**Figure 11:**
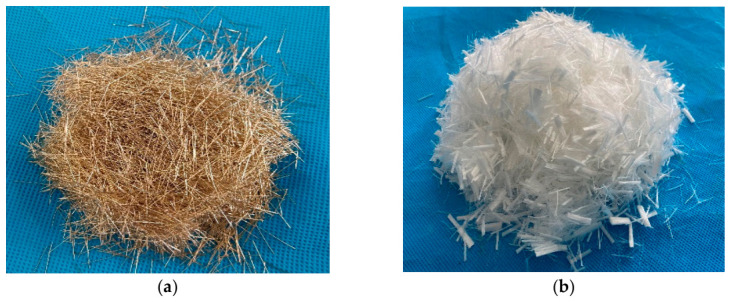
Macroscopic morphology of (**a**) steel fiber and (**b**) PVA fiber.

The aqueous epoxy resin emulsion (F0704) and aqueous epoxy resin curing agent (F0705) were employed in this study, which were supplied by Shenzhen Jitian Chemical Industry Co., Ltd. (Shenzhen, China). The technical parameters of epoxy resin emulsion are summarized in Table 2, while the parameters of aqueous epoxy resin curing agent are listed in Table 3. Nano-SiO_2_ was incorporated into the system, with its properties detailed in Table 4. A silicon defoamer (active content > 30%) was used to minimize air entrainment. Cementitious components included P.I 42.5 cement (compliant with JC/T 2381-2016 [43]) and quartz sand (1.42~2.85 mm particle size, Gongyi Yuanheng Water Purification Material Factory, Gongyi, China) [44]. A polycarboxylate-based water reducer (Subote New Material Co., Ltd., Nanjing, China) with a water reduction rate of 27% was employed to enhance workability. Hybrid fibers consisting of copper-coated steel fibers (diameter: 0.21 mm, length: 13 mm, tensile strength: 2750 MPa) and PVA fibers (Kuraray China Co., Ltd., Shanghai, China) were utilized for reinforcement. The parameters of the PVA fibers are provided in Table 5, and the macroscopic morphologies of both fiber types are illustrated in Figure 11.

### 4.2. Mixing and Preparation of Specimens

The optimal mixture ratio of nano-SiO_2_-modified repair mortar was obtained based on the DL/T 5126-2021 [45] and studies by other researchers, including a water–cement ratio of 0.52, a cement–sand ratio (mass ratio between sand and cementitious materials, including cement and nano-SiO_2_) of 2.2, a polymer–cement ratio (the mass ratio between solid of epoxy resin emulsion and cementitious materials) of 9%, and a nano-SiO_2_ content (replacing cement at an equal mass substitution rate) of 1.5% [46,47,48]. In addition, the epoxy resin emulsion needs to be employed with the curing agent, with the mass ratio of 2. The addition of a defoamer was set at 1.2% of the epoxy resin solid quality to reduce the harmful influence of extensive air in the matrix. The fiber addition was expressed as a volumetric fraction of the matrix. It should be noted that the control variable method was adopted in this research, so the single-factor test was conducted to investigate the influence of steel fiber or PVA fiber on the mechanical properties of HF-NERM. To be specific, the addition of PVA fiber was 0%, 0.3%, 0.6%, 0.9% and 1.2%. The incorporation of steel fiber was 0%, 0.4%, 0.8%, 1.2% and 1.6%. Finally, details about the mix proportion of HF-NERM are illustrated in Table 6.

**Figure 12:**
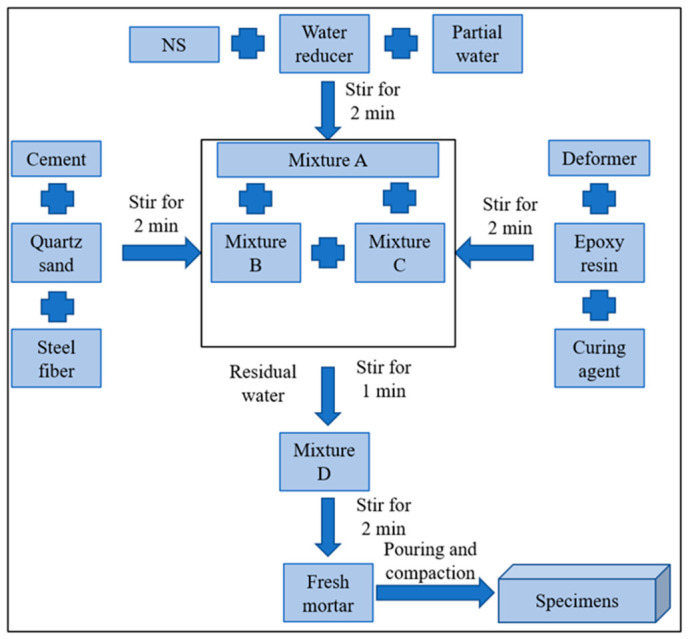
Preparation procedure of hybrid fiber-reinforced nano-SiO_2_-modified repairing mortar.

HF-NERM was prepared following the guidelines of DL/T 5126-2021. Prior to mixing, nano-SiO_2_ was dispersed in water via ultrasonication for 30 min to ensure homogeneous distribution within the matrix. A detailed schematic of the preparation process is provided in Figure 12. After casting, the specimens were subjected to curing for 28 days under controlled conditions (20 ± 2 °C, ≥95% relative humidity). To systematically evaluate the effects of steel and PVA fibers on mechanical performance, nine sample groups were designed. To isolate the effects of PVA fibers, the steel fiber content was held constant at 1.2% volume throughout the study. Conversely, the PVA fiber content was fixed at 0.9% volume when analyzing the influence of steel fibers.

### 4.3. Methods

#### 4.3.1. Fluidity Experiment

According to DL/T 5126-2021, the fluidity of HF-NERM was evaluated, utilizing a standard slump cone with dimensions of φ25 mm (top) × φ50 mm (bottom) × 150 mm (height). Three parallel specimens were prepared for each mixture proportion to ensure statistical reliability. Fresh HF-NERM was poured into the slump cone in two equal layers. Each layer was compacted with 15 radial strokes using a standard concrete vibrator, moving from the edge to the center. Following vibration, the slump cone was vertically lifted in a controlled manner. The slump value was determined by measuring the vertical difference between the stabilized surface of the HF-NERM and the original cone height using a calibrated ruler.

#### 4.3.2. Mechanical Properties Experiments

The cube compressive strength test and the post-flexural test were performed to evaluate the compressive properties. Three parallel specimens were prepared for each mixture proportion in all mechanical tests. The test instrument was a 2000 kN computer-controlled compression testing machine, and the specimens with dimensions of 70.7 mm × 70.7 mm × 70.7 mm were prepared for the cube compressive strength test. Flexural strength tests were performed following the GB/T 17671-2021 [49], and prism specimens (40 mm × 40 mm × 160 mm) were subjected with a 300 kN cement mortar flexural testing machine [50]. After flexural failure, the two fractured prism halves were clamped in a compression fixture and tested for post-flexural compressive strength using the same 2000 kN machine employed for cube compressive tests. The splitting tensile strength was determined based on JGJ/T 70-2009 [51] and supplementary methodologies from prior studies. Cubic specimens (70.7 mm × 70.7 mm × 70.7 mm) identical to those used for compressive strength testing were loaded diametrically using the aforementioned 2000 kN compression testing machine.

To quantity the impact of fibers on mechanical properties of HF-NERM, the hybrid effect index ($\alpha_{sp}$) was adopted for further analysis [29]. The hybrid effect index is used to quantitatively evaluate whether the combination of PVA fiber and steel fiber produces a positive synergistic effect. A value greater than 0 indicates that the hybrid-fiber system outperforms the single-fiber system and presents an obvious synergistic strengthening effect. This index directly reflects the rationality of fiber matching and provides a quantitative basis for the optimal mix design of HF-NERM. The calculation of $\alpha_{sp}$ is illustrated in Equations (1) and (2). When $\alpha_{sp}$ < 0, the incorporation of hybrid fiber was considered a negative influence. On the contrary, the addition of hybrid fiber expressed a positive effect on the mechanical properties when $\alpha_{sp}$ > 0.

(1) $$\alpha_{sp}=\frac{S_{sp}-(S_{s}\varphi_{s}+S_{p}\varphi_{p})}{S_{s}\varphi_{s}+S_{p}\varphi_{p}}$$

(2) $$\varphi_{s}+\varphi_{p}=\frac{V_{s}}{V_{sp}}+\frac{V_{p}}{V_{sp}}=1$$

where $S_{sp}$$S_{s}$ and $S_{p}$ are the mechanical performance of HF-NERM influenced by hybrid fiber, steel and PVA fiber, respectively. $V_{s}$, $V_{p}$ and $V_{sp}$ are the volume of steel fiber, PVA fiber and hybrid fiber, respectively. $\varphi_{s}$ and $\varphi_{p}$ are the proportions of steel fiber and PVA fiber.

#### 4.3.3. Flexural Toughness Experiments

**Figure 13:**
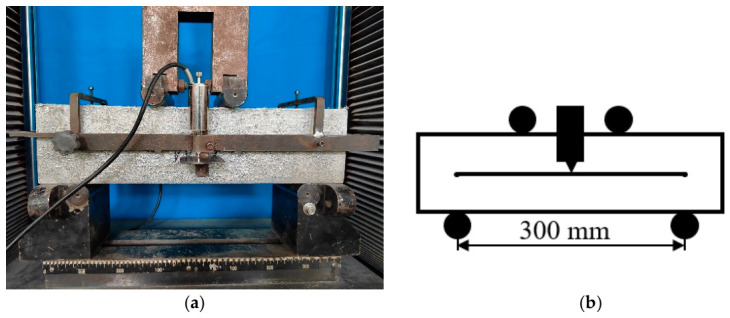
Diagram (**a**) and schematic diagram (**b**) of flexural toughness experimental.

In accordance with ASTM-C1018 [52], flexural toughness tests were performed using a four-point loading setup. Three parallel specimens were prepared for each mix proportion to ensure statistical validity. Nine mix proportions were tested, each comprising three prismatic specimens (100 mm × 100 mm × 100 mm). A 1000 kN electro-hydraulic universal testing machine was utilized for loading. Load and displacement data were synchronously recorded by using a load cell (50 kN capacity, ±1 N accuracy) and linear variable differential transformers (LVDTs, sensitivity: 0.35 mV/mm) for mid-span deflection measurement. Prior to testing, the support span was adjusted to 300 mm. Specimens were positioned with their molded side surfaces resting on the supports. Two LVDTs were symmetrically mounted at mid-span to monitor deflection, with averaged values reported. The test protocol included preloading, zero calibration and monotonic loading. A schematic of the experimental setup and a representative test configuration are depicted in Figure 13.

**Figure 14:**
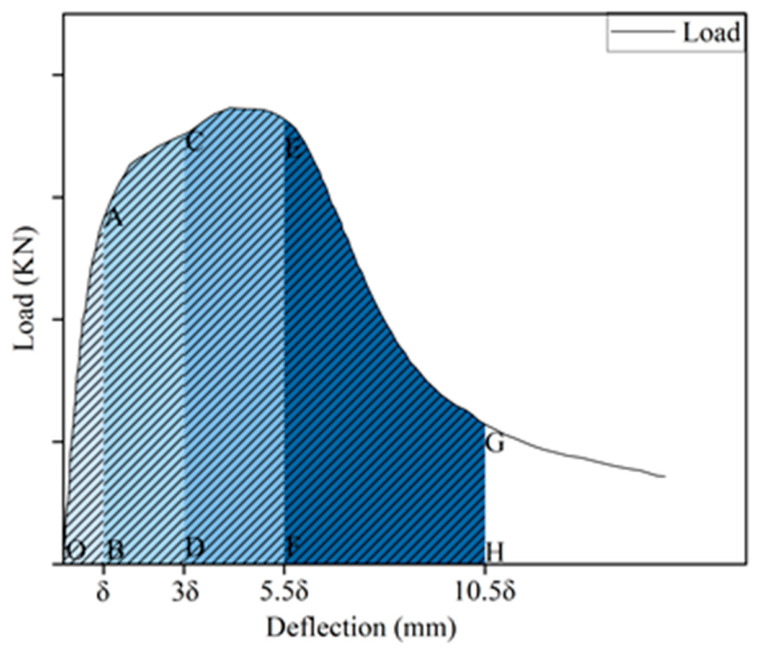
Schematic diagram of ASTM method.

The most employed flexural toughness evaluation was ASTM-C1018. According to this method, the toughness index was calculated from the ratio between the area under the load–deflection curve up to a given multiple of the first-crack deflection and the area up to the first-crack point itself. Rooted in elastoplastic mechanics, it effectively quantifies fiber contributions to the post-cracking behavior of cementitious composites. A typical schematic diagram of the ASTM method is illustrated in Figure 14. Point δ was defined as the deflection when the first crack appeared. Four deflections, *δ*, 3*δ*, 5.5*δ*, 10.5*δ*, were selected and labeled as points B, D, F and H, respectively. The area under the load–deflection curve corresponding to segments OAB, OACD, OAEF and OAGH was calculated as $\Omega_{\delta }$, $\Omega_{3\delta }$, $\Omega_{5.5\delta }$ and $\Omega_{10.5\delta }$, respectively. The flexural toughness indices I5, I10 and I20 were calculated using Equations (3)–(5), respectively [52]. Flexural toughness indices reflect the energy absorption capacity of the material under flexural load. Larger values indicate stronger deformation resistance and higher toughness after cracking. These indices are key indicators for evaluating the anti-cracking, anti-seismic, and durability performance of repair mortar in practical engineering. In addition, residual strength factors were determined using Equations (6) and (7), respectively, which were employed to quantify the post-cracking residual load-bearing capacity of the material. Residual strength factors characterize the post-cracking load-bearing capacity and ductility retention of HF-NERM. Higher values mean the material can still maintain stable bearing performance after large deformation, which is critical for preventing sudden brittle failure of repaired structural members. Through these quantitative indices, the mechanical performance, deformation characteristics, and failure toughness of hybrid fiber-reinforced NERM can be comprehensively and accurately evaluated, providing a reliable theoretical and methodological reference for engineering application.

(3) $$I_{5}=\frac{\Omega_{3\delta }}{\Omega_{\delta }}$$

(4) $$I_{10}=\frac{\Omega_{5.5\delta }}{\Omega_{\delta }}$$

(5) $$I_{20}=\frac{\Omega_{10.5\delta }}{\Omega_{\delta }}$$

(6) $$R_{5,10}=20(I_{10}-I_{5})$$

(7) $$R_{10,20}=10(I_{20}-I_{10})$$

#### 4.3.4. Bonding Property Experiments

**Figure 15:**
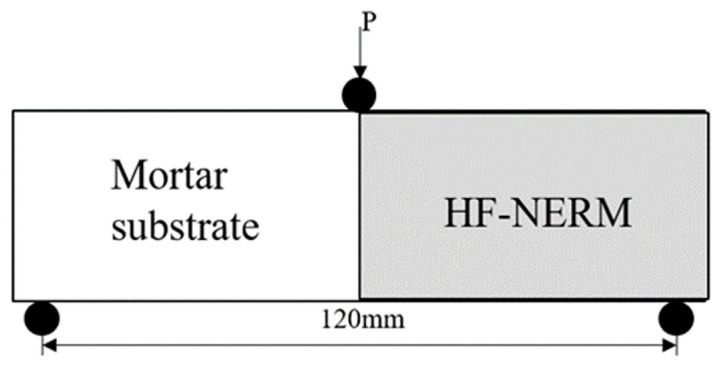
Schematic diagram of bonding properties experimental.

The bond property of repair mortars (40 mm × 40 mm × 160 mm) was determined following the JC/T 2381-2016 and the methods researched by other researchers [3]. Six parallel specimens were prepared for each mixture proportion to ensure statistical reliability. The interfacial flexural tensile strength was adopted to evaluate the bonding properties of HF-NERM. Prior to testing, the bonding specimens underwent a 28-day curing period. The interface flexural tensile strength was then determined by a standard flexural strength test, with the experimental setup illustrated in Figure 15.

#### 4.3.5. Morphological Observation

The microstructure morphology of HF-NERM was characterized using scanning electron microscopy ((SEM, KYKY-EM6900, Beijing Zhongke Instrument Co., Ltd., Beijing, China). The observation surface was retained in its natural fractured state, while other surfaces were polished to ensure planarity. Specimens were ultrasonically cleaned in anhydrous ethanol to stop the hydration stage. Before testing, samples were oven-dried for 24 h to eliminate residual moisture. To enhance electron conductivity, dried specimens were sputter-coated with a gold layer. The prepared specimens were then transferred to the SEM vacuum chamber for high-resolution imaging.

## Figures and Tables

**Table 1:** Parameters of flexural properties of HF-NERM.

Test Number	Initial Cracking Load (kN)	Peaking Load (kN)	Ultimate Deflection (mm)
P0S12	14.03	18.02	1.38
P03S12	14.47	20.13	1.54
P06S12	14.71	23.18	1.72
P09S12	14.82	24.22	2.05
P12S12	14.73	23.04	2.44
P09S0	13.84	16.42	1.29
P09S04	14.28	20.78	1.39
P09S08	14.60	22.81	1.70
P09S16	15.01	24.73	1.77

**Table 2:** Technical parameters of epoxy resin emulsion.

Solid Content (%)	Solid Epoxy Resin Content	pH	Specific Gravity	Viscosity (25 °C, MPa·s)
50 ± 3	400–800	2–7	1.01–1.08	<1000

**Table 3:** Properties of epoxy resin curing agent.

Solid Content (%)	Solid Epoxy Resin Content	pH	Specific Gravity	Viscosity (25 °C, MPa·s)
44 ± 2	260 ± 60	8–11	1.00–1.08	>2000

**Table 4:** Properties of nano-SiO_2_.

Mean Particle Size (nm)	Content (%)	Specific Surface Area (m^2^/g)	Apparent Density (g/L)	pH
30	99.5	220	55	6

**Table 5:** Properties of PVA fiber.

Diameter (μm)	Length (mm)	Elongation at Break (%)	Elastic Modulus (GPa)	Tensile Strength (MPa)
40	12	41	16.5	1560

**Table 6:** Proportion of HF-NERM.

Group	Cement	Quartz Sand	Water	Polymer–Cement Ratio	NS	PVA Fiber	Steel Fiber	Defoaming Agent	Water Reducer
	kg/m^3^	kg/m^3^	kg/m^3^	%	%	%	%	%	kg/m^3^
P0S12	571.3	1276	208.6	9	1.5	0	1.2	0	14.5
P03S12	571.3	1276	208.6	9	1.5	3	1.2	1.2	14.5
P06S12	571.3	1276	208.6	9	1.5	6	1.2	1.2	14.5
P09S12	571.3	1276	208.6	9	1.5	9	1.2	1.2	14.5
P12S12	571.3	1276	208.6	9	1.5	12	1.2	1.2	14.5
P09S0	571.3	1276	208.6	9	1.5	9	0	1.2	14.5
P09S04	571.3	1276	208.6	9	1.5	9	0.4	1.2	14.5
P09S08	571.3	1276	208.6	9	1.5	9	0.8	1.2	14.5
P09S16	571.3	1276	208.6	9	1.5	9	1.6	1.2	14.5

Note: P09S12 represents the HF-NERM with 0.9% PVA fiber and 1.2% steel fiber.

## Data Availability

The raw data supporting the conclusions of this article will be made available by the authors on request.
